# Bioactive Potential and Chemical Composition of Green Peel of Dominico-Hartón (*Musa* AAB) Ethanolic Extract

**DOI:** 10.3390/molecules31213883

**Published:** 2026-10-28

**Authors:** Alison Vanesa Benavides-Garzón, Bryan Emanuel Cadena-Hincapie, Juan Camilo Guerrero-Ospina, Patricia Landázuri

**Affiliations:** 1 Programa de Doctorado en Ciencias Biomédicas, Facultad de Ciencias de la Salud, Universidad del Quindío, Armenia 630001, Quindío, Colombia; avbenavides@uniquindio.edu.co (A.V.B.-G.); jcguerrero@uniquindio.edu.co (J.C.G.-O.); 2 Programa de Biología, Facultad de Ciencias Básicas y Tecnologías, Universidad del Quindío, Armenia 630001, Quindío, Colombia; bryancadenahincapie@gmail.com; 3 Programa de Medicina, Programa de Doctorado en Ciencias Biomédicas, Facultad de Ciencias de la Salud, Universidad del Quindío, Armenia 630001, Quindío, Colombia

**Keywords:** plantain, *Musa*, endothelial cells, peel, Dominico-Hartón

## Abstract

*Musa* AAB is a key crop in Quindío (Colombia), and its peel—an agro-industrial byproduct—has been shown to contain polyphenols with antioxidant properties that are important for cardiovascular health. The objective was to characterize the ethanolic extract of the green peel (EEGPP) of the Dominico-Hartón variety (*Musa* AAB) harvested in Quindío and to evaluate its antioxidant activities and its effect on cell viability in HMEC-1 cells. The EEGPP was obtained by maceration. Total phenols, flavonoids, and glycosides were quantified by spectrophotometry; polyphenols were identified and quantified by liquid chromatography coupled with mass spectrometry. Antioxidant activity was evaluated using the DPPH and TABARS assays, and cell viability was assessed using the SRB assay. The extract yield was 1.37%; the extract had a higher content of flavonoids and phenols. Several polyphenols were identified in high concentrations: 3,5-dihydroxybenzoic acid, rutin, p-coumaric acid, and vanillic acid. The extract exhibited dose-dependent antioxidant activity and increased cell viability at low concentrations after 24 h of exposure in HMEC-1 cells. These findings suggest that the EEGPP from *Musa* AAB is a potential source of bioactive compounds with antioxidant activity and that it promotes the viability of HMEC-1 endothelial cells, which encourages further investigation into its applicability.

## 1. Introduction

It is very important to distinguish between plantains (cooking bananas) and bananas (dessert bananas), both of the genus *Musa* but with significantly different phytochemical profiles [1,2]; in this research we used *Musa paradisiaca* (plantains), genomic variety AAB Dominico-Hartón, this variety has not been fully characterized in its secondary metabolites. Plantain (*Musa paradisiaca* L.) is a monocotyledonous plant belonging to the Musaceae family and represents one of the oldest and most essential crops for global food security, particularly in tropical regions of Africa, Asia, and America [1,2]. In Colombia, plantain production is a socioeconomic pillar with more than 400,000 ha planted and an annual production exceeding 3.5 million tons [3]. Within this industry, the Dominico-Hartón variety stands out as the predominant cultivar in the coffee-growing region and the department of Quindío, accounting for 65% of national production [4].

Despite its economic importance, the production chain generates approximately 23 million tons of waste annually (leaves, pseudostems, and peels), of which 38% is not used in any way [5,6]. This accumulation of residual biomass is a source of environmental pollution and a significant source of greenhouse gases (61% of organic emissions in landfills), as well as acting as a reservoir for phytopathogens in the field [3]. However, these byproducts, especially the peel, are rich sources of biologically relevant secondary metabolites, such as phenolic compounds and flavonoids [2,7]. These substances have antioxidant properties linked to the prevention of cardiovascular diseases (CVDs), which are the leading cause of global mortality [8].

The pathophysiology of CVDs is closely linked to oxidative stress (OS), a process that induces endothelial dysfunction and promotes the formation of oxidized low-density lipoproteins (oxLDLs), and the endothelium loses its protective role, a key element in atherogenic progression. Therefore, the selective uptake of reactive oxygen species (ROS) by phyto-protective compounds emerges as a promising therapeutic strategy to protect the endothelium and prevent LDL oxidation [9,10]. Under this pharmacological approach, it is imperative to validate the biological safety of new plant extracts through in vitro cytotoxicity assays.

In the field of vascular research, the human microvascular endothelial cell line (HMEC-1) is a critical reference model, as it retains the phenotypic characteristics of normal human endothelium [11]. Consequently, studying the activity of *Musa* AAB extracts in HMEC-1 models allows their therapeutic potential to be characterized and their cellular safety to be ensured prior to future clinical applications.

Regarding the plantain, most research studies focus on the edible part, but place little or no emphasis on the chemical composition of its peels, or on how they can be used and turned into medicines or adjuvants for various diseases.

Based on this, the objective of this study was to chemically characterize the extract of the green peel of Dominico-Hartón (*Musa* AAB) for antioxidant and cytotoxic activities in human microvascular endothelial cells (HMEC-1).

## 2. Results

### 2.1. Percentage Yield of Extraction by Maceration and Secondary Metabolite Quantifications

The obtained ethanolic extract of the green peel plantain Dominico-Hartón (EEGPP) appeared as a dark green powder, with an extraction yield of 1.37%, with a total phenolic content of 67.50 mg GAE/g dw (Figure 1A), a concentration of 992.13 mg CE/g dw (Figure 1B) and 32.07 mg GE/g dw (Figure 1C). These values highlight the antioxidant potential of the byproducts of this variety and indicate that flavonoids are one of the major fractions within the identified secondary metabolites.

### 2.2. Quantification of Phenolic Compounds and Flavonoids in the EEGPP by Liquid Chromatography–Triple Quadrupole Mass Spectrometry (LC-QqQ)

The results obtained for the EEGPP of Dominico-Hartón (*Musa* AAB) are presented in Table 1 (negative polarity) and Table 2 (positive polarity).

### 2.3. Biological Activity of the Ethanolic Extract of Green Peel of Dominico-Hartón (Musa AAB)

#### 2.3.1. Antioxidant Activity

The antioxidant activity of the EEGPP was quantified using a DPPH assay. The results show, as expected, that the higher the concentration of the extract, the greater its capacity to inhibit the free radical (Figure 2).

The analysis of variance (ANOVA) revealed statistically significant differences (0.0005 and 0.049) in the percentage of inhibition between 0.3, 0.7 and 1 respectively, but no significant differences were observed between the concentrations of 0.3 mg/mL and 0.7 mg/mL.

#### 2.3.2. Assay of Substances Reactive to Thiobarbituric Acid (TBARS)

The assessment of antioxidant capacity using the TBARS assay (Figure 3) revealed an inversely proportional relationship with respect to the concentration of the plant extract being evaluated. The highest protective activity was recorded at the lowest concentration tested, 0.3 mg/mL, reaching 86%. In contrast, when the extract concentration was increased to 0.7 mg/mL and 1.0 mg/mL, a progressive reduction in the percentage of antioxidant activity was observed, decreasing to 68.55% and 53.66%, respectively. This decrease was statistically significant when comparing the 0.3 mg/mL concentration to the 1.0 mg/mL concentration (*p* < 0.01). Uric acid was used as the reference standard. The obtained absorbance value (0.0037 ± 0.0011) reflects an inhibition of basal lipid peroxidation, confirming that the chemical conditions of the assay (reagents, temperature, and incubation times) functioned optimally and reproducibly.

#### 2.3.3. Effect of the Ethanolic Extract of Green Peels on the HMEC-1 Cell Line Viability

The viability analysis of the EEGPP in the HMEC-1 cell line revealed a biphasic behavior dependent on exposure time. At 24 h, none of the evaluated concentrations (0.3, 0.7, and 1.0 mg/mL) showed cytotoxic effects; in fact, they exceeded the viability of the untreated control. The highest cell viability (measured as total protein biomass) was observed with 0.3 mg/mL, reaching a viability of 123.60%, relative to the untreated control, followed by the concentrations of 0.7 mg/mL (112.2%) and 1.0 mg/mL (108.8%). In contrast, the positive control (10% DMSO) validated the assay with a significant reduction (36.83%) in cell viability. However, after 48 and 72 h of exposure, a significant time- and dose-dependent reduction in cellular viability was established in all treatments, as viability decreased to critical levels: 27.22% (0.7 mg/ml), 22.51% (1.0 mg/mL), and 16.58% (0.3 mg/mL) at 72 h. These findings suggest that, while the extract increases cellular viability in acute exposures, prolonged contact (>24 h) induces a gradual process of cell death, possibly mediated by nutrient depletion or the accumulation of secondary metabolites at levels toxic to the endothelium (Figure 4).

#### 2.3.4. The Mean Cellular Inhibitory Concentration (IC50)

The mean cellular inhibitory concentration (IC50) could only be determined for the 48 h exposure time, reaching a value of 0.42 mg/mL. At 24 h, it was not possible to calculate the IC50 due to increased cell viability (negative values). At 72 h, no IC50 value was obtained within the evaluated range, as all concentrations induced reduction in cell viability than 70%, exceeding the 50% inhibition threshold.

## 3. Discussion

The yield of the EEGPP was 1.37%. This value is within the range reported in the literature for plantain peel extracts (*Musa paradisiaca)* using a variety of solvents; Widoyanti et al. [12] obtained a yield of 1.27% using ethyl acetate. Although the yields are similar, the metabolite contents in the extract may vary due to the polarity conditions of the solvent, also depending on factors such as the plantain variety, the extraction method and the growth conditions of the crop.

The phytochemical screening of extracts in this study showed that Dominico-Hartón peels contain various phytochemicals like alkaloids, flavonoids, and phenols. The total phenol content (67.60 mg GAE/g dw) in the EEGPP exceeds the values reported by Widoyanti et al. [12], who obtained ±59 mg GAE/g dw using similar protocols. This phytochemical content confirms that ethanol has an optimal hydrophilic–lipophilic balance for the solubilization of phenolic compounds, surpassing the efficiency of less polar solvents such as ethyl acetate or purely aqueous systems [13]. The variability observed compared to other studies can be attributed to physicochemical and methodological factors, since the solubility of phenols is conditioned by the polarity of the solvent and thermal stability during extraction. Factors such as exposure to oxygen and light can induce the oxidative degradation of these metabolites, altering their final quantification [12,14], or also by biological determinants such as the biosynthesis of phenolic compounds in the peel of *Musa* spp., responding to soil and climate conditions, the state of ripeness, and cellular defense mechanisms.

Overall, the high concentration of phenols found in the Dominico-Hartón variety supports its potential value as a source of natural antioxidants for biomedical applications.

The flavonoid content recorded in the EEGPP (992.13 mg CE/g dw) is significantly higher than the values documented in previous studies with ethanolic extracts of the same species, such as those by Oyeyinka & Afolayan (641.53 mg CE/g dw) [15], and Laeliocattleya et al. [16] (416.08 mg CE/g dw). This marked superiority suggests a robust phytochemical profile in the Dominico-Hartón plantain variety.

This was corroborated by liquid chromatography–mass spectrometry results, which identified and quantified different phenolic compounds and flavonoids in the extract, with the highest concentration being 3,5-dihydroxybenzoic acid, followed by rutin, among others, as can be seen in Table 1 and Table 2. Hydroxybenzoic acids are related to salicylic acid and salicin, the first compounds isolated with pharmacological activity [17]. The phenolic compound 3,5-dihydroxybenzoic acid (α-resorcylic acid) activates hydroxycarboxylic acid receptors, leading to a reduction in lipolysis in adipocytes and possible improvements in the blood lipid profile [17]. On the other hand, the flavonoid rutin or quercetin-3-rutoside is one of the most common forms of quercetin in natural sources; it has shown pharmacological benefits in chronic diseases such as hypertension and hypercholesterolemia, as in the work of Bhandary et al. [18], who observed that rutin had beneficial effects against hemodynamic alterations associated with cardiac damage due to ischemia–reperfusion in isolated rat hearts, mainly due to its antioxidants.

Our metabolic analysis of polyphenols in Musa peels compares various analytical techniques and their quantitative results. Regarding rutin, in this study using LC-QqQ determined a concentration of 170 µg/g, while Passo et al. (2014) reported 242.2 µg/g using HPLC-DAD and HPLC-ESI-HR-MS [19]. This variation is due to the detection principles of each technique. HPLC-ESI-HR-MS is used to identify compounds through precise mass determination and structural elucidation, while LC-QqQ is based on monitoring specific precursor-product transitions (MRMs), offering greater sensitivity and selectivity for targeted metabolite quantification. In general, however, both studies agree that the peel represents an important source of rutin.

Regarding other metabolites, when comparing the LC-QqQ results in Dominico-Hartón plantains with the LC-ESI-QTOF-MS/MS profile in *Musa acuminata* (Bashmil et al., 2021), p-hydroxybenzoic acid was found to be the main metabolite in LC-ESI-QTOF-MS/MS [20], but was detected at low concentrations with LC-QqQ; meanwhile, p-coumaric acid was predominant in Dominico-Hartón (LC-QqQ), compared to low concentrations recorded with LC-ESI-QTOF-MS/MS. Finally, epicatechin was detected at low concentrations in both analytical methodologies.

These differences reflect, firstly, the high metabolic plasticity of the genus *Musa*, regulated by the variety, ripening, and environmental conditions, and secondly, by the extraction procedures.

In relation to glycoside content in the extract at 1 mg/mL was 32.07 mg EG/g ES, whereas Frebriyossa et al. [21] determined that the green peel of *Musa paradisiaca* contains 38.15 mg of these compounds, which is not far from what was found in this study. Glycosides are compounds found in various plants and have been reported to have biological activities such as antioxidants and antimicrobials [2].

Given the above findings, EEGPPs were evaluated for their ability to quench DPPH radicals. As shown in Figure 2, the extracts’ DPPH radical scavenging activity is dose-dependent, with statistically significant difference (*p* < 0.0006), although the capacity to trap DPPH radicals was below 70%; when compared with extracts obtained in other works, this was similar to that described by Singh et al. for the extract of *Musa balbisiana* peel [22].

The difference in DPPH radical quenching between the extracts may be due to the plantain variety and the nature of the phenolic compounds contained in each extract, which can interact in various ways to reduce free radicals [15]; it may also be due to the nature of the solvent used.

Several studies [12,15] have demonstrated a positive correlation between phenolic and flavonoid contents and antioxidant activity, noting that it is stronger for flavonoids; thus, different phytochemical analyses of *Musa paradisiaca* peel extracts have identified antioxidant secondary metabolites such as epicatechin, gallocatechin, rutin, myricetin, and naringenin [23,24]. Furthermore, recent research has shown that higher extract concentrations are associated with increased levels of phenolics and flavonoids, resulting in enhanced antioxidant activity, thereby indicating a dose-dependent effect [12,15].

The assessment of antioxidant capacity using the TBARS assay showed that the plant extract exhibited its maximum activity at the lowest concentration tested (0.3 mg/mL), with a progressive and statistically significant decrease at higher concentrations (0.7 and 1.0 mg/mL). Although a direct dose–response relationship is typically expected in in vitro assays, the loss of antioxidant effectiveness at high concentrations is a widely documented phenomenon in extracts rich in phytochemicals [25].

This nonlinear response can be explained by the concentration-dependent pro-oxidant effect characteristic of various phenolic compounds and flavonoids. In the presence of transition metals such as iron (Fe-EDTA)—an essential component of the Fenton system used in this assay—high concentrations of polyphenols can act as strong reducing agents, reducing Fe^3+^ ions to Fe^2+^. This acceleration of the iron redox cycle increases the generation of hydroxyl radicals (HO*) and the subsequent degradation of the matrix, exceeding the extract’s capacity to scavenge these radicals [26,27].

Similar behaviors have been described in extracts derived from agricultural byproducts of the Musaceae family. Studies such as those by González-Montelongo et al. (2010) [28] and Azofeifa et al. (2014) [29] on *Musa* spp. peel demonstrated that the high density of reducing phytochemicals at elevated concentrations promotes pro-oxidative reactions in vitro, reaching an antioxidant threshold at low-to-intermediate doses before declining.

Additionally, the contribution of kinetic–chemical saturation phenomena or spectral interference at 532 nm caused by the self-aggregation of pigments and fat-soluble compounds at 1.0 mg/mL—which alters the absorbance of the MDA-TBA adduct [30]—cannot be ruled out. Taken together, the findings suggest that the extract possesses a significant window of antioxidant efficacy at low doses (0.3 mg/mL), optimally preventing oxidative damage without triggering the pro-oxidant effects associated with higher doses.

This study also evaluated the effect of EEGPP on the HMEC-1 cell line. After 24 h of exposure, no cytotoxic effects were observed; instead, an increase in cell viability was detected at low concentrations, suggesting a possible hormetic or adaptative response. However, after 48 and 72 h, cell viability progressively decreased, suggesting a possible hormetic or adaptative response and indicating a time- and dose-dependent cytotoxic effect.

This unexpected pattern may be explained by physical–chemical and biological factors characteristic of complex botanical extracts. At higher concentrations (1.0 mg/mL), hydrophobic or polyphenolic constituents can undergo self-aggregation or partial micro-precipitation in aqueous culture media, effectively reducing the bioavailable fraction of active cytotoxic compounds in solution compared to lower, fully solubilized concentrations (0.3 mg/mL). Alternatively, higher extract concentrations may induce early cytostasis or cellular aggregation that physically preserves protein biomass detectable by SRB, whereas fully soluble lower doses promote initial metabolic acceleration (as observed at 24 h) followed by rapid nutrient exhaustion and cell death during unrenewed static exposure. Given the inherent limitations of total biomass assays, this nonlinear response warrants further experimental verification using single-cell viability and membrane-integrity assays (e.g., flow cytometry with Annexin V/PI and LDH release).

Given the lack of literature under the same conditions, our findings are compared with studies on similar species (Euterpe oleracea, Cynara cardunculus, Grateloupia longifolia) that reported initial increases in cell viability at low concentrations, where low extract concentrations promoted proliferation, while higher concentrations reduced cell viability [31,32]. Ghosh et al. (2017) [33] evaluated *Musa paradisiaca* root extracts on HUVEC endothelial cells and observed a dose-dependent cytotoxic effect. The higher the concentration of the extract, the greater the cell mortality. This biphasic pattern is characteristic of hormesis, a phenomenon in which low doses trigger adaptive or protective cellular responses, whereas high doses induce toxicity [34].

The increased proliferation observed at 24 h may be attributed to polyphenols such as quercetin and epicatechin, which can activate proliferative signaling pathways like PI3K and modulate redox balance [35]. Over time, however, the accumulation of reactive oxygen species (ROS) and metabolic byproducts likely exceeded the cellular antioxidant capacity, leading to oxidative stress and subsequent cell death.

Several authors have shown that natural antioxidants derived from plant extracts, used alone or in combination, can be a solution to counteract the loss of endothelial functionality and increase the viability of endothelial cells; thus, it has been shown that *Morus alba* L., *Origanum majorana* L. and *Salvia fruticosa* Mill. have the ability to improve endothelial functions such as relaxation and vasodilation through various mechanisms [36,37,38].

A limitation of the present study is that cell viability was assessed exclusively via the SRB assay, which reflects overall cellular protein biomass. While this approach is well-established for preliminary cytotoxicity screening, it cannot discriminate between cytostatic effects, apoptosis, or necrosis. Future investigations incorporating flow cytometry (Annexin V/PI), LDH release, and specific apoptotic markers will be necessary to elucidate the exact cell-death mechanisms involved at 48 and 72 h.

In summary, this study addresses a specific gap in the phytochemical literature by presenting the first detailed LC-MS characterization of individual polyphenols in the green peel of the Dominico-Hartón cultivar (*Musa* AAB) from Quindío, Colombia. The selected HMEC-1 model is appropriate and scientifically sound for evaluating the extract’s bioactivity and vascular safety. Furthermore, this study aligns with the priorities of sustainability and the circular economy by repurposing an agro-industrial byproduct with significant regional impact, supported by an experimental design that evaluates multiple concentrations in both radical scavenging (DPPH) assays and cell viability assays.

## 4. Materials and Methods

### 4.1. Material Plant Collection

Green plantain peels (GPPs) from the Dominico-Hartón (*Musa* AAB) variety were used, which were supplied by the company Platanillos Crocantes del Quindío (La Tebaida, Colombia).

#### 4.1.1. Peel Selection Criteria

The selection criteria for GPP included: only the Dominico-Hartón variety (*Musa* AAB) from Quindío, taken on the same day; peels that were ripe, showed signs of mechanical damage, or had the presence of phytopathogens and chemicals were excluded.

#### 4.1.2. Preparation of Plant Material

The GPPs were transported to the Biochemistry and Genetics Laboratory of Universidad del Quindío; they were washed with neutral soap, tap water and subsequently with distilled water. They were then subjected to a drying process at 50 °C for 3 days. Once dry, the peels were ground in a food processor to a fine powder.

#### 4.1.3. Obtaining the Ethanolic Extract from the Green Peel of Dominico-Hartón (*Musa* AAB) by Maceration

A total of 600 g of pulverized plant material was added to 1200 mL of 96% ethanol; the mixture was left under constant stirring at room temperature for 10 days, as recommended for adequate diffusion of secondary metabolites in dense or lignocellulosic matrices (such as peels) at room temperature [39]. At the end of the maceration process, the mixture was filtered, the supernatant was saved and the solid residue was eliminated. The supernatant was concentrated by rotary evaporation and was subsequently dried in an oven at 37 °C for 2 days. Finally, the extract was stored at −4 °C until further use.

#### 4.1.4. Percentage Yield of the Green Peel Extract of Dominico-Hartón (*Musa* AAB)

Once the extract was obtained by maceration with 96% ethanol, the percentage yield was calculated with Equation (1).

(1) $$\text{\% }\mathrm{Yield}=\frac{\mathrm{Wf}}{\mathrm{Wi}}\times 100$$

where Wi is the initial weight and Wf is the final weight.

### 4.2. Characterization of the Ethanolic Extract of the Green Peel of Dominico-Hartón (Musa AAB)

#### 4.2.1. Secondary Metabolite Quantifications

##### Quantification of Total Phenols by the Folin–Ciocalteu Method

This method was slightly modified by our research group. In summary, 500 µL of the extract was taken at a concentration of 1 mg/mL and then the Folin–Ciocalteu reagent (1:1) was added. The mixture was left to stand for 2 min, then 20% of a *Na*_2_*CO*_3_ solution was added. After stirring the mixture, it was incubated for an hour and a half. Finally, the absorbances were measured at 765 nm in a UV/Vis spectrophotometer (Thermo Electron Corporation-Genesys 10UV, Thermo Electron Corporation, Waltham, MA, USA). Three independent tests were performed, each in triplicate. Gallic acid was used as a standard and distilled water as a negative control. Quantification was performed using a calibration curve of gallic acid at different concentrations. The results were expressed in milligrams of gallic acid equivalent per gram of extract (mg EAG/g extract) [40].

#### Flavonoid: Content Quantification

The colorimetric reaction method of 2,4-dinitrophenylhydrazine and AlCl_3_ was used. In summary, 250 µL of the extract at 1 mg/mL was taken; 1250 µL of distilled water and 75 µL of 5% sodium nitrite were added; it was left to stand for 6 min; combined with 90 µL of 10% AlCl_3_, then 500 µL of 0.1 M NaOH and 335 µL of distilled water; homogenized; and the absorbances were read at 500 nm. Three independent tests were performed, each in triplicate. Quantification was carried out using a calibration curve of catechin at different concentrations, and the results were expressed in milligrams of catechin equivalent per gram of extract (mg EC/g extract) [41].

#### Glycoside Quantification

To determine polysaccharides, the phenol–sulfuric acid method was used, according to López-Legarda et al. (2017) [42], modified by our research group. An amount of 500 µL of the extract at 1 mg/mL was taken, then 250 µL of 5% phenol and 1250 µL of 95% H_2_SO_4_ were added. It was then incubated at room temperature for 15 min, and finally the absorbances were measured at 490 nm, using distilled water as a blank. Three independent tests were performed, each in triplicate. For this test, glucose was used as a standard for the preparation of the calibration curve using different concentrations, and the results were expressed in milligrams of glucose equivalent per gram of dry extract (mg GE/g extract).

#### Phenolic and Flavonoids Quantification by HPLC

Metabolite extraction

Free phenolic compounds were extracted using 11.5 mg of sample in 1 mL of 80% (*v*/*v*) ethanol. The sample was shaken at 200 rpm for 10 min at 25 °C and then centrifuged at 5000 g for 10 min at the same temperature. The resulting pellet was subjected to alkaline hydrolysis by adding 400 μL of 4 M NaOH and treating it with ultrasound for 90 min at 40 °C. Next, acid hydrolysis was performed by adjusting the pH to 2 with concentrated HCl, followed by centrifugation at 2000 g for 5 min at 25 °C. The supernatant was resuspended in 1 mL of ethyl acetate and centrifuged again.

The extracts corresponding to free and bound phenolic compounds were evaporated in a SpeedVac concentrator for 4 and 1 h, respectively. They were then resuspended in 120 μL of a mixture of methanol, acetonitrile, and Milli-Q water (2:5:93, *v*/*v*/*v*).

Analysis of the samples by liquid chromatography–triple quadrupole mass spectrometry (LC-QqQ):

The sample was analyzed using an Agilent Technologies 1260 liquid chromatography system coupled with a 6470 triple quadrupole mass analyzer (LC-QqQ), Agilent Technologies, Santa Clara, CA, USA, with electrospray ionization. An amount of 3 μL of the sample was injected into a C18 column (InfinityLab Poroshell 120 EC-C18, 2.1 × 150 mm, 2.7-micron, Agilent Technologies, Santa Clara, CA, USA) at 30 °C, using a composed phase mobile of A (0.1% *v*/*v* formic acid in Milli-Q water) and B (0.1% *v*/*v* formic acid in acetonitrile), at a constant flow rate of 0.4 mL/min. The chromatographic elution gradient began with 2% Phase B, then gradually increased to 30% Phase B at minute 10, and subsequently rose to 98% at minute 20. This condition was maintained for an additional 2 min. Subsequently, the gradient returned to the initial conditions, with a 5 min re-equilibration period. Mass spectrometry detection was performed in MRM (Multiple Reaction Monitoring) mode, using an ESI source in both negative and positive modes. This targeted analytical approach allowed the monitoring of specific transitions from precursor ions to product ions for the compounds of interest. Analyte identification was based on comparison with analytical reference standards, in accordance with the identification criteria established during the analysis.

Quantification of the compounds was performed using semi-quantification based on the area of the chromatographic peaks. The results were expressed as µg/g.

### 4.3. Biological Activity of the Ethanolic Extract of the Green Peel of Dominico-Hartón (Musa AAB)

#### 4.3.1. Evaluation of Antioxidant Activity

The antioxidant activity was evaluated by the 2,2-difenil-1-picrilhidrazilo (DPPH) radical capture method, described by Alinezhad et al. [43]; briefly, 20 µL of each extract concentration (0.3, 0.7, 1 mg/mL) was taken, 1980 µL of DPPH 0.05 mM was added, it was left to incubate at room temperature and in darkness for 30 min, then the absorbances were measured at 517 nm in a spectrophotometer (Thermo Electron Corporation-Genesys 10UV, Thermo Electron Corporation, Waltham, MA, USA) for three independent tests, each in triplicate. A calibration curve was carried out with trolox at different concentrations and the percentage of inhibition of the DPPH radical was determined using Equation (2).

(2) $$\%\mathrm{Inhibition}=\frac{{(A}_{C}-\mathrm{As})}{A_{C}}\times 100$$

where Ac is the absorbance of the control and As is the absorbance of the sample.

#### 4.3.2. Test for Substances Reactive to Thiobarbituric Acid (TBARS)

A peripheral blood sample was obtained via venipuncture from an apparently healthy volunteer donor after the donor had signed an informed consent form in accordance with applicable ethical guidelines. The blood was collected in tubes without anticoagulant and centrifuged at 3.800 rpm for 10 min to separate the serum, which was immediately used for the assays.

The effect of the extracts on hydroxyl radicals was evaluated by determining the inhibition of damage to sodium benzoate. In separate reaction tubes, 100 μL of serum was mixed with 100 L of the extract at different final concentrations (0.3, 0.7, and 1.0 mg/mL). Subsequently, 490 μL of phosphate-buffered saline (PBS, 10X), 500 μL of sodium benzoate, 200 μL of the {Fe-EDTA} complex, and 200 μL of hydrogen peroxide were added. The mixture was incubated at 37 °C for 1 h.

Upon completion of the incubation, the reaction was stopped by adding 1.000 μL of 20% (*v*/*v*) acetic acid and 1000 μL of 2-thiobarbituric acid (TBA). The mixtures were heated in a water bath at 100 °C for 10 min. After cooling, the absorbance was measured at 532 nm using a spectrophotometer. Serum without extract was used as the blank; distilled water as the negative control; and uric acid as the reference standard (1 mmol/L).

Antioxidant activity was calculated using the following formula:

$$\mathrm{AOA}=\frac{\left(C_{\mathrm{UA}}\right)(K-A)}{K-U_{A}}$$

where:

*C_UA_*: Uric acid concentration (1 mmol/L).

*A*: Absorbance of the sample.

K: Absorbance of the control.

*U_A_*: Absorbance of the uric acid standard.

#### 4.3.3. HMEC-1 Cell Line Viability Evaluation

The HMEC-1 cell lines were provided by the University of Antioquia. The cells were cultured following the methodology described by Carvajal et al. [44]. A 3% maintenance medium was used, consisting of 47 mL of DMEM medium with L-glutamine, 1.5 mL of fetal bovine serum, 0.5 mL of penicillin–streptomycin (P/S), and 0.5 mL of non-essential amino acids. Subsequently, the cells were incubated at 37 °C in a humidified atmosphere containing 5% CO_2_. Afterwards, HMEC-1 cells were seeded in 96-well microplates at a density of 20.000 cells per well, using 200 µL of test medium (3% maintenance medium), and allowed to grow for 24 h until reaching approximately 90% confluence. Once confluence was achieved, the plates were distributed according to the exposure times of 24, 48, and 72 h.

The dry extract was re-dissolved in sterile DMEM to prepare stock solutions and diluted in maintenance medium (DMEM with 3% FBS) immediately prior to treatment.

Treatments were then applied as follows: blank (3% medium only), positive control (10% dimethyl sulfoxide), negative control (3% medium + cells), and the test treatments with three concentrations of the extract under study (0.3, 0.7, and 1 mg/mL).

Treatments were administered as a single static dose at time zero, and culture media were not renewed during the 24 h, 48 h, or 72 h incubation periods.

After each exposure period, 50 µL of trichloroacetic acid (TCA, 50%) was added to each well, and the plates were incubated for 1 h at 4 °C. The wells were then washed with tap water and allowed to dry for 24 h. Subsequently, 100 µL of sulforhodamine B (SRB, 0.4%) was added and incubated for 30 min. The wells were then washed four times with 1% acetic acid and allowed to dry again. Finally, 200 µL of Tris base solution (10 mM, pH 10.5) was added, and absorbance was measured at 490 nm using a spectrophotometer (Thermo Electron Corporation-Genesys 10UV, Thermo Electron Corporation, Waltham, MA, USA).

Controls: To eliminate potential optical background or interference from colored extract components, cell-free blank wells containing extract in medium at corresponding concentrations (without cells) were subjected to identical TCA fixation, SRB staining, washing, and solubilization procedures. Absorbance values from these cell-free blanks were subtracted from treated cell values.

The results were expressed as cell viability percentage (% cell viability) determined by SRB, which corresponds to the proportion of live cells in a sample compared to the total number of cells present; therefore, the cell viability percentage was calculated using Equation (3):

(3) $$\%\;\mathrm{cell}\;\mathrm{viability}=\frac{\mathrm{Average}\;\mathrm{absorbance}\;\mathrm{of}\;\mathrm{treated}\;\mathrm{cells}}{\mathrm{mean}\;\mathrm{absorbance}\;\mathrm{of}\;\mathrm{control}\;\mathrm{cells}}\times 100$$

### 4.4. Statistical Analysis

All assays were performed in triplicate, and the results are expressed as the mean ± standard error of the mean (SEM). The normality of the data was assessed using the Shapiro–Wilk test, with the exception of the cytotoxicity assay, for which the Kolmogorov–Smirnov test was used. Homoscedasticity was verified using Bartlett’s test for parametric data and the Fligner–Killeen test for nonparametric data. To determine differences among the concentrations evaluated, a one-way analysis of variance (ANOVA) was used, followed by Tukey’s post hoc test for multiple comparisons. In the phenol quantification assay, given the non-normal distribution of the data, the nonparametric Kruskal–Wallis test was used, supplemented with Dunn’s post hoc test. The influence of treatments and controls on vascular endothelial cell viability was evaluated using a Generalized Linear Model (GLM). A Gamma error distribution with a logarithmic link function was assumed, defining the percentage of cell viability as the response variable. The samples: (controls *n* = 12 and treatments *n* = 16) and exposure time (24, 48, and 72 h) were included as fixed factors in three independent experiments. Multiple comparisons in the GLM were determined using Tukey’s post hoc test.

GLM modeling was performed using R software (version 4.4.2). All other statistical analyses, including simple linear regression analyses for the calibration curves and the creation of graphs, were performed using GraphPad Prism (version 8.0.1.244). In all cases, a *p*-value of <0.05 was considered statistically significant.

## 5. Conclusions

This study provides, for the first time, a detailed phytochemical characterization and an assessment of the bioactive potential of the ethanolic extract from the green peel of the Dominico-Hartón banana cultivar (*Musa* AAB), an agricultural byproduct with significant socioeconomic impact in the Quindío region of Colombia. The extract obtained is notable for its high concentration of flavonoids and total phenolics. Through LC-QqQ analysis, a distinctive polyphenolic profile was identified, dominated by 3,5-dihydroxybenzoic acid, rutin, p-coumaric acid, and trans-cinnamic acid.

In terms of functional activity, the extract exhibited concentration-dependent antioxidant capacity in the DPPH assay, as well as notable protection against lipid peroxidation as measured by TBARS, reaching its maximum efficacy at low concentrations.

Assessment in HMEC-1 cells revealed a biphasic or hormetic response: acute exposure (24 h) to moderate concentrations (0.3 mg/mL) increase cellular viability, whereas prolonged treatment (>24 h) induces dose-dependent cytotoxicity (IC_50_ of 0.42 mg/mL at 48 h).

Taken together, these findings confirm that *Musa* AAB peel is a promising source of antioxidant phytochemicals capable of preserving vascular endothelial viability within specific ranges. Furthermore, this study supports the agro-industrial valorization of these plant residues, transforming a regional environmental liability into a sustainable raw material with potential pharmaceutical, cosmetic, or food applications. Future research is needed to isolate specific bioactive fractions and elucidate the molecular mechanisms underlying endothelial proliferation.

## Figures and Tables

**Figure 1:**
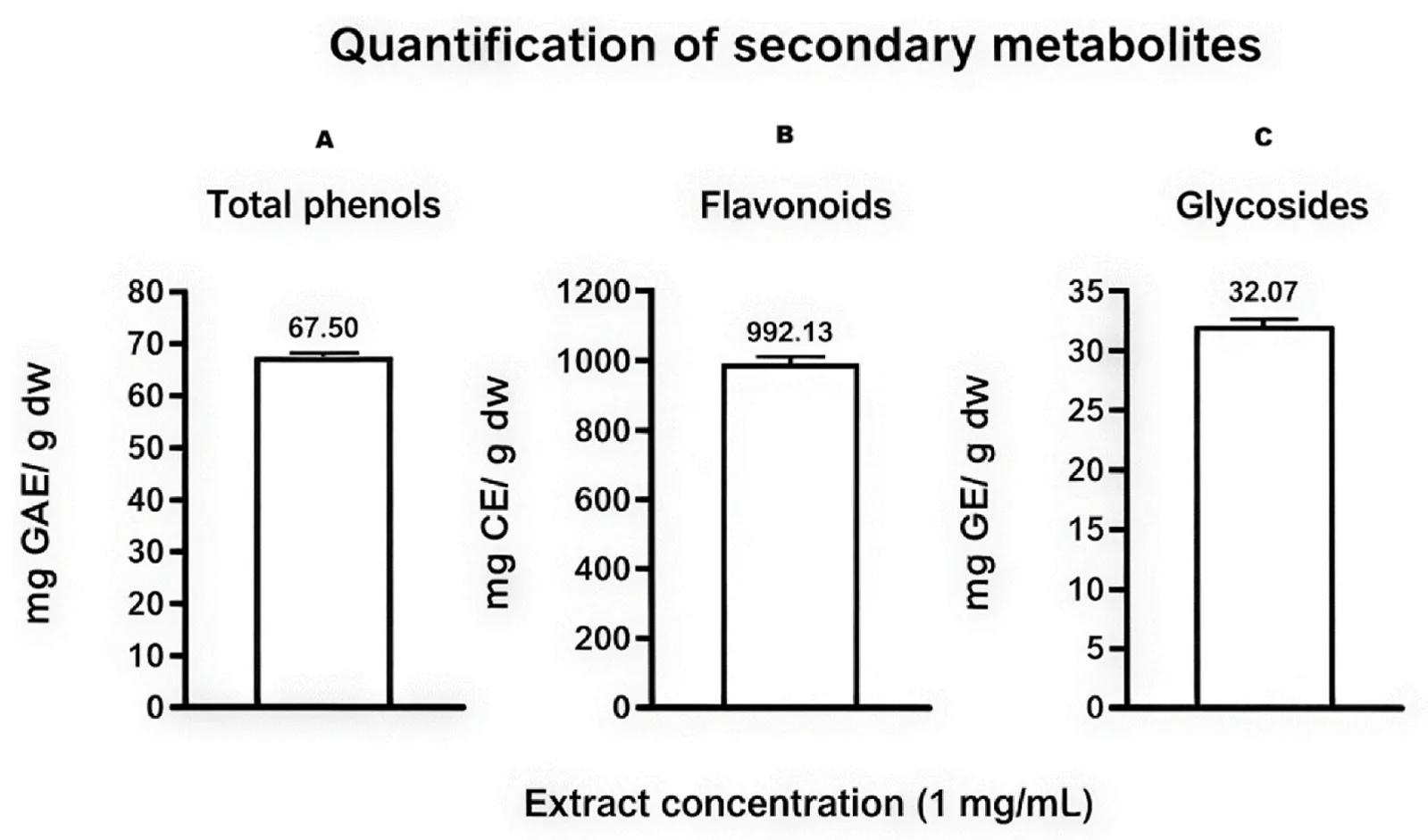
Spectrophotometric quantification of secondary metabolites in the extract of green peel from Dominico-Hartón (*Musa* AAB). (**A**) Total phenol content; values are expressed in milligrams of gallic acid equivalent per gram of dry extract (mg GAEg/g dw). (**B**) Flavonoid content; values are expressed in milligrams of catechin equivalent per gram of dry extract (mg CE/g dw). (**C**) Glycoside content; values are expressed in milligrams of glucose equivalent per gram of dry extract (mg GE/g dw). Error bars correspond to SEM bars.

**Figure 2:**
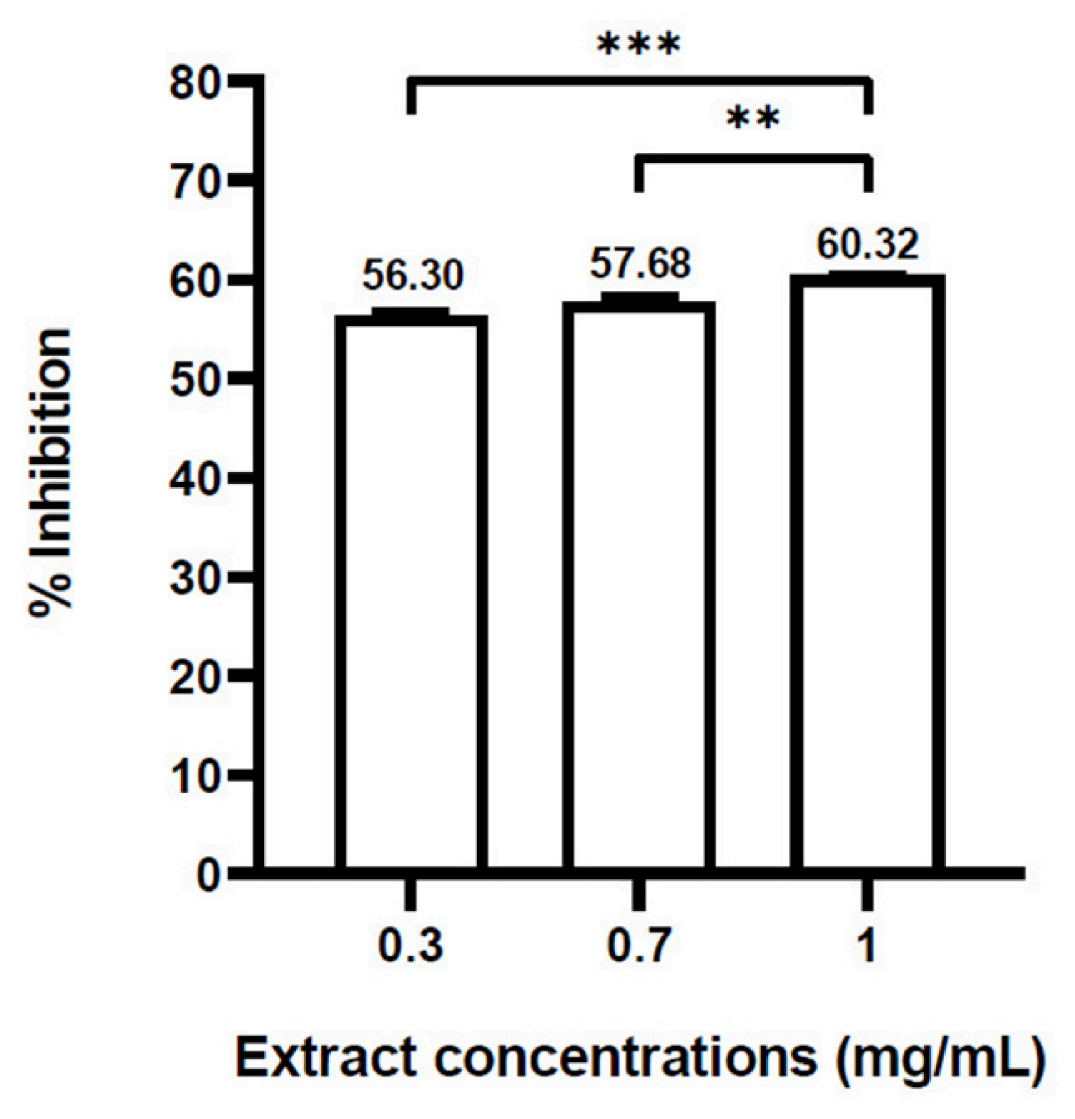
Antioxidant activity of the green peel extract of Dominico-Hartón (*Musa* AAB) using the DPPH assay. The results are expressed as percentage of free radical inhibition. The error bars represent the standard error of the mean. ** = *p* < 0.01; *** = *p* < 0.001 (*n* = 3).

**Figure 3:**
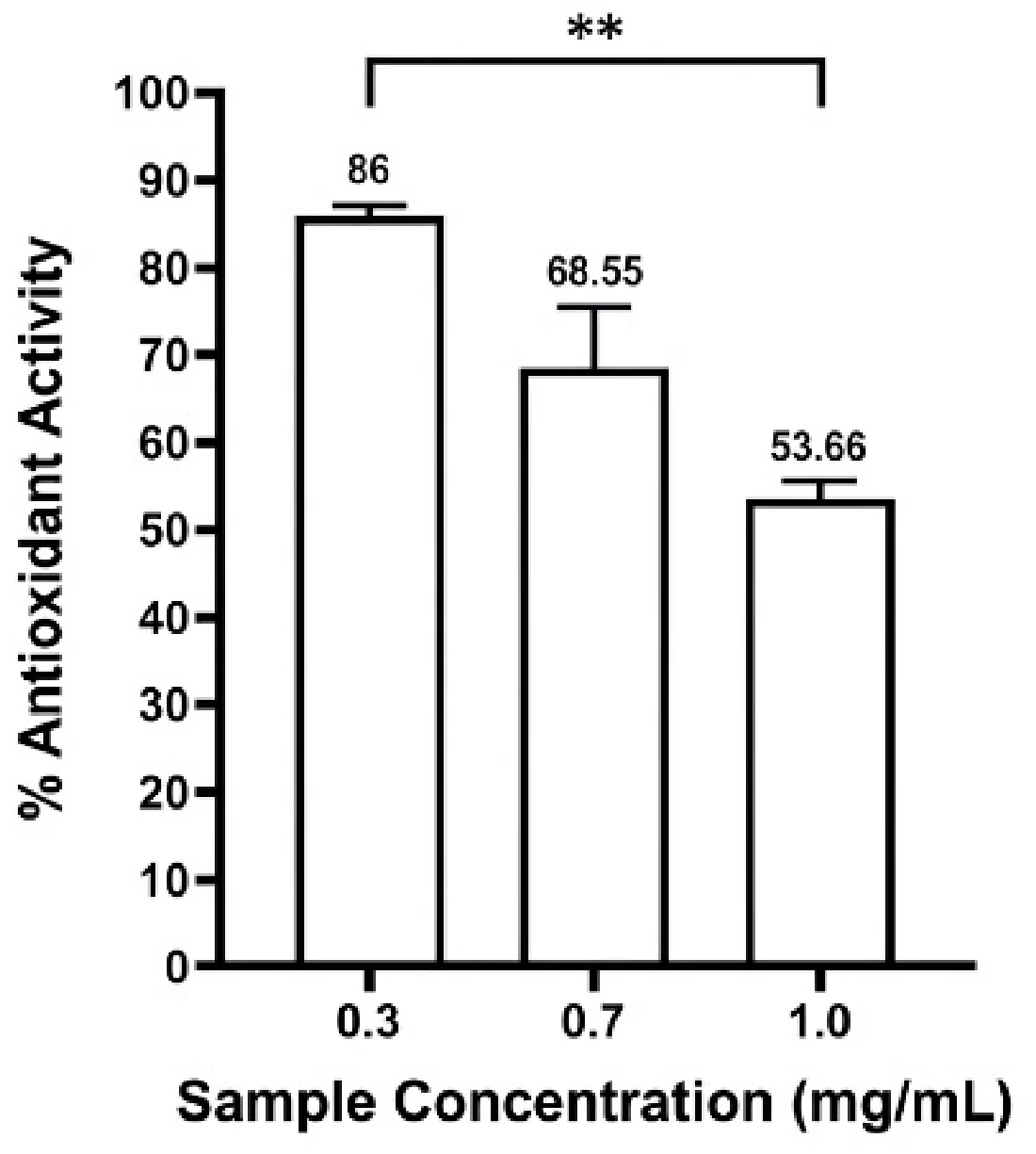
Antioxidant capacity of the plant extract assessed using the TBARS assay. The percentages of antioxidant activity at concentrations of 0.3, 0.7, and 1.0 mg/mL are presented. Uric acid (1 mmol/L) was included as a reference standard and absorbance (0.0037 ± 00.11) was used to calculate the percentage of inhibition of the extracts. Data are expressed as the mean ± standard error of the mean (SEM) from three independent experiments (*n* = 3). The bars and asterisks indicate statistically significant differences between the indicated treatments (** *p* < 0.01, one-way ANOVA followed by Tukey’s test).

**Figure 4:**
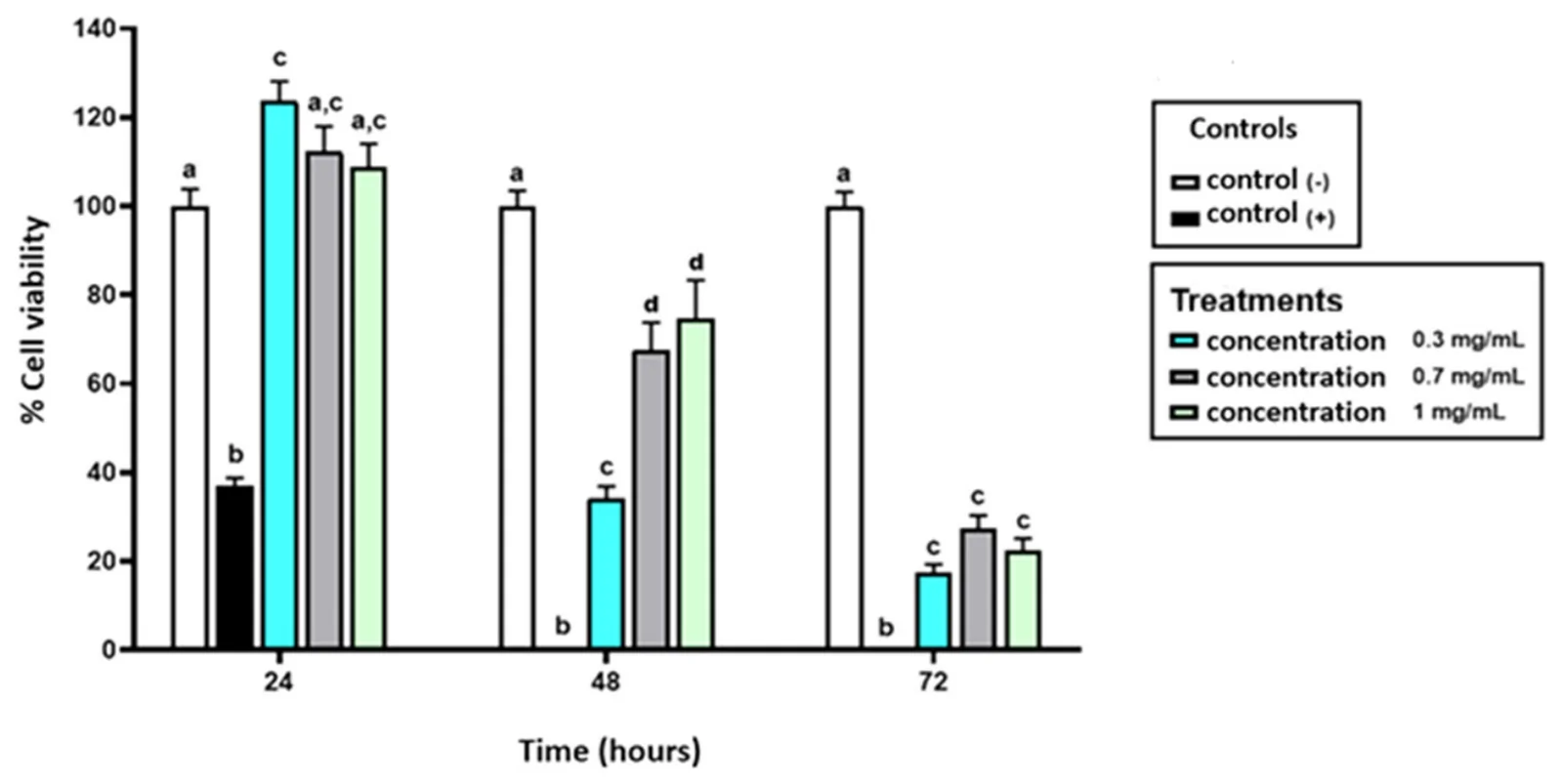
Effect of the ethanolic extract of green peels from Dominico-Hartón on the cell viability of HMEC-1 lines exposed to different exposure times (24 h, 48 h, and 72 h). The concentrations of the extract to be evaluated were 0.3, 0.7, and 1 mg/mL. Cytotoxicity was evaluated using the SRB colorimetric method. The values are expressed as a percentage of cell viability (%). The error bars correspond to standard error bars. Repeated letters indicate that there are no significant differences between groups, while letters that are not repeated between groups indicate that there are significant differences between groups (Tukey’s test, <0.05).

**Table 1:** Quantification of phenolic compounds and flavonoids in EEGPP of Dominico-Hartón (*Musa* AAB) in negative polarity.

Name	RT (min)	Transition	Sample (µg/g)
**Gallic acid**	**3.075**	**169.0 to 125.0**	**7.02**
3,5-Dihydroxybenzoic acid	4.810	152.9 to 108.9	228
2,3,4-Trihydroxybenzoic acid	4.945	168.9 to 150.9	2.10
Catechol	5.620	109.0 to 108.0	2.20
4-Hydroxybenzoic acid	6.230	137.0 to 93.0	74.5
Gentisic acid	6.293	153.0 to 108.0	1.01
Chlorogenic acid	6.551	353.0 to 191.0	0.750
4-Acetocatechol	6.905	151.0 to 108.0	2.75
Terephthalic acid	6.980	165.0 to 121.0	11.9
Vanillic acid	7.023	167.1 to 151.9	80.4
3-Methoxyhydroxinnamic acid	7.180	179.0 to 135.0	8.53
Caffeic acid	7.200	179.0 to 135.0	8.97
Dihydroxybenzoic acids (2,3-, 2,4-, 2,6-)	7.227	153.0 to 109.0	6.65
3-Hydroxybenzoic acid	7.320	137.0 to 93.0	0.381
Syringic acid	7.379	197.0 to 182.0	12.0
(−)-Epicatechin	7.710	289.0 to 245.0	0.254
4-Methylcatechol	8.600	123.0 to 108.0	0.310
p-Coumaric	8.607	163.0 to 119.0	99.9
Hydroferulic acid	8.843	195.0 to 136.0	5.42
Rutin	9.240	609.0 to 300.1	170
Ferulic acid	9.250	193.0 to 134.0	75.2
Sinapic acid	9.289	223.0 to 193.0	3.06
(+)-Taxifolin	9.682	303.0 to 285.0	0.316
Diosmin	10.448	607.0 to 299.1	0.0203
Hesperidin	10.613	609.0 to 301.0	0.132
Salicylic acid	10.727	137.0 to 93.0	4.35
Rosmarinic acid	10.980	359.0 to 161.0	0.055
Myricetin	11.064	316.9 to 151.0	0.361
Phloridzin	11.223	435.0 to 273.0	0.273
Baicalin	11.669	444.9 to 269.0	0.0109
Luteolin	12.736	284.9 to 133.0	0.0181
Quercetin	12.810	300.9 to 121.0	0.0917
Morin	12.830	301.0 to 121.0	0.100
(+/−)-Naringenin	13.830	270.9 to 150.9	0.0661
Kaempferol	14.078	284.9 to 93.1	0.0840
Diosmetin	14.116	299.0 to 284.1	0.0236
Caffeic acid phenethyl ester	16.192	283.0 to 135.0	0.0126
Biochanin A	16.233	282.9 to 268.0	0.00405

**Table 2:** Quantification of phenolic compounds and flavonoids in EEGPP of Dominico-Hartón (*Musa* AAB) in positive polarity.

Name	RT (min)	Transition	Sample (µg/g)
Coniferyl alcohol	8.467	163.0 to 131.0	0.160
Sinapyl Alcohol	8.550	193.0 to 161.0	0.272
Sinapic acid	9.297	225.0 to 207.0	2.13
o-Anisic Acid	9.320	153.0 to 135.0	0.849
Veratric acid	9.530	183.0 to 139.0	2.27
trans-Cinnamic acid	13.094	149.0 to 131.0	134

RT = retention time.

## Data Availability

The original contributions presented in this study are included in the article. Further inquiries can be directed to the corresponding author.
